# Pharmaceutical Notices

**Published:** 1847-07

**Authors:** William Procter


*Pharmaceutical Notices.* By William Procter, Jr.

*Donovan's Solution.*—The formula for Donovan's solution, as published by its author, and of which a copy is found in the United States Dispensatory, presents so complex an appearance in the way of fractions of a grain, that many apothecaries, not possessed of a delicate balance and weights are deterred from making it.

Another objection is, that a loss of iodine is occasioned by the exposure rendered necessary during the trituration of the three elements with the alcohol during the time that elapses before the combination is effected. The mercury rapidly acquires its dose of iodine, whilst the arsenic, which combines less readily, is liable to all the deficiency occasioned by vaporization. That this occurs is evident from a small quantity of arsenic left undissolved by the distilled water. The proportions in Donovan's formula are 6.08 grains of arsenic, 14.82 grains of mercury, and 49 grains of iodine, which are not exactly in atomic relation. In order that the preparation should be composed of one equivalent of each iodide, the numbers should be 5.5 arsenic, 14.82 mercury, 46.09 iodine, so that there is one-tenth of an equivalent of the arsenical iodide in excess. It is hardly probable that any sensible variation would be observable in the medicinal action of the solution if the iodides were used in equivalent proportion; and as their combining number is the same, the preparation of the solution may be much simplified.

The aggregate of the solid contents of half a pint of Donovan's solution is 70 grains, which is composed of 33⅓ grains of biniodide of mercury and 36⅔ grains of sesqui-iodide of arsenic. If the formula was made to require 35 grains of each iodide, and half a pint of water, it would be greatly simplified, and could be made extemporaneously. The addition of hydriodic acid to the solution is unnecessary in this case, and its design in the formula of Donovan is doubtless to supply any deficiency due to loss of iodine in the operation.

The following formula, closely resembling that of Donovan in the proportions, avoids the fractions of a grain:

Take of Sesqui-iodide of arsenic, 36 grs:
" Biniodide of mercury, 34 "
" Distilled water, half a pint.

Triturate the two iodides with half an ounce of the water until they combine and dissolve, and then add the rest of the water, and filter.

The sesqui-iodide of arsenic is easily prepared. One part of metallic arsenic is reduced to an impalpable powder, intimately mixed with five parts of iodine by trituration, then introduced into a small flask or thin vial, and the mixture very gently heated until liquefaction occurs. The vessel should be nearly full, so as to prevent the formation of much iodine vapour, and enable the operator to bring the fused iodide in contact with all parts of it, and include any iodine that may have sublimed on the sides. If, on cooling, the contents of the vial assume a reddish yellow colour and crystallize on the sides of the vial, and no iodine odour is apparent, the operation is finished. The vessel is then fractured and the iodide removed.

If the arsenic and iodine are pure, and the process skilfully conducted, nearly the whole of the arsenic is combined, and the compound is quite pure enough for making Donovan's solution without sublimation.

The metallic arsenic should be bright and lustrous, and the iodine crystallized and free from water. The ordinary cobalt or fly stone, which is metallic arsenic, is often pure enough for pharmaceutical use.

*Atropia.*—The activity of good extract of belladonna, leaves little to desire on the part of the practitioner in the internal exhibition of this drug, the dose being quite small, yet in its external application, with a view to the dilatation of the pupil of the eye, or as an application in neuralgia, the extract is so disagreeable an application that several European surgeons have sought to avoid the staining effect on the skin by resorting to the active principle, atropia, in an isolated condition. In the February number of the *Journal de Pharmacie et de Chimie*, it is stated that "among the new remedies employed by M. Berard in the treatment of diseases of the eye, *atropia* should be placed in the first rank. This alkaloid, which can now be obtained from some pharmaceutists, possesses some incontestable advantages in certain cases over belladonna itself.

"The greatest of these advantages is certainly the extreme rapidity with which it affects the dilatation of the pupil even in very minute quantity, as for instance by a solution containing one grain in one hundred grains of distilled water.

"Another advantage appreciated by the patient is the fact that the use of atropia avoids all the smearing and staining of the face, so characteristic of the ordinary mode of applying the extract; and which creates so great a repugnance to its use."

According to the Dublin Quarterly Journal (November, 1845, page 553,) "Dr. Wilde of that city employs the solution of atropia of these strengths:

No. 1. Atropia grs. j. distilled water f.ʒj. diluted alcohol gtts. iij.
No. 2. Atropia grs. ij. distilled water f.ʒj. diluted alcohol gtts. iij.
No. 3. Atropia grs. iij. distilled water f.ʒj. diluted alcohol gtts. iij.

The alkaloid is rendered soluble by a drop of nitric acid, and the spirit is added to make the solution keep.

A single drop of No. 1 placed on the conjuctiva of the lower lid causes dilatation of the pupil in a healthy eye in from five to fifteen minutes."

The best process yet published for atropia is that of Mein, noticed in the United States Dispensatory, and in which the root is the subject of treatment with alcohol, lime, etc. As we cannot get belladonna root here, the only available source is the best extract, which, independent of the large amount of inert matter to get rid of, is too expensive to be employed with that view. Those who incline to make the experiment, however, may proceed by treating the extract with warm water until all the soluble portion is dissolved, filtering out the chlorophylle and albumen, evaporating the solution to a syrup, dissolving this in alcohol, and filtering, if necessary, and then proceeding as in Mein's process with lime, acid, etc.

Belladonna leaves contain a large quantity of dark colouring matter, which renders the recent juice claret coloured, and is the chief cause of the staining. A solution of extract of belladonna precipitated with subacetate of lead, the excess of lead carefully with sulphuric acid, and the solution filtered from the sulphate of lead, carefully evaporated to four times the weight of the extract, no doubt would answer all the purposes of the solution of atropia.

*Syrup of Orange Peel.*—Those who are in the habit of preparing the syrup of orange peel according to the United States Pharmacopœia, know that it is strongly disposed to fermentation, and rapidly loses its agreeable qualities in warm weather. When made by the following formula it is more aromatic and preserves readily.

Two ounces of the recently dried peel of the sweet orange is reduced to powder and lixiviated with a mixture of two parts of alcohol and one of water, until six fluid ounces are obtained. This tincture is then poured over and mixed well with 32 ounces (av.) of sugar in coarse powder, and spread on paper until the alcohol has evaporated. When this is accomplished, the aromatized sugar is made into syrup with 16 fluid ounces of water, merely carrying the heat to ebullition in a covered vessel, straining and bottling hot. Prepared in this way, syrup of orange peel has a fine amber colour, and the orange taste in a marked degree.—*Am. Journ. of Pharm.*

